# *‘They are targeted as fun and better for you than smoking’*: Australian parents’ opinions about the normalisation of vaping for children and young people

**DOI:** 10.1177/17579139251319668

**Published:** 2025-04-01

**Authors:** S Thomas, S McCarthy, H Pitt, B Freeman, G Arnot, M Daube

**Affiliations:** Institute for Health Transformation, Faculty of Health, Deakin University, Locked Bag 20000, Geelong, VIC 3220, Australia; School of Population Health, Curtin University, Perth, WA, Australia; Institute for Health Transformation, Faculty of Health, Deakin University, Geelong, VIC, Australia; Institute for Health Transformation, Faculty of Health, Deakin University, Geelong, VIC, Australia; School of Public Health, University of Sydney, Sydney, NSW, Australia; Faculty of Health, Institute for Health Transformation, Deakin University, Geelong, VIC, Australia; Faculty of Health Sciences, Curtin University, Perth, WA, Australia

**Keywords:** parents, vaping, children, normalisation, public health

## Abstract

**Introduction:**

Parents can play an important role in shaping youth vaping attitudes and behaviours, and are important stakeholders in advocating for policy reforms to protect the health of children and young people. Few studies have qualitatively investigated parents’ perceptions of the factors that contribute to the normalisation of vaping for children and young people. This study aimed to understand the range of factors that parents attributed as playing a role in shaping the normalisation of vaping for young people, and the risks that these products pose to the health and wellbeing of young people.

**Methods:**

An online qualitative survey was conducted in December 2022 with *n* = 455 Australian parents of at least one child aged 11–17 years. Open text questions interpreted for this article investigated parents’ perceptions about whether they thought vapes were normalised for young people; the reasons young people were interested in vaping; and the impact of marketing and novel products on vaping attitudes and behaviours in young people. Data were analysed using reflexive thematic analysis.

**Results:**

Parents were clear that vaping was becoming increasingly normalised for young people. They attributed this to four key factors: (1) Peer influences (particularly through school settings); (2) The increased accessibility and availability of vapes in community settings; (3) The role of marketing and novel product design; and (4) The perception that vapes were a healthier alternative to cigarettes or were not harmful.

**Discussion and Conclusion:**

Understanding parents’ views is an important part of developing public health responses to harmful products. Parents were highly aware of, and concerned about, the increased normalisation of vaping for children, and should be engaged by health organisations to support and advocate for policy reform.

## Introduction

Vapes (or e-cigarettes) cause serious health risks to young people and have become a significant public health threat.^1^ Vapes often contain nicotine and other toxic substances that lead to adverse health effects.^2–4^ Globally, there is a growing prevalence of vaping among young people,^5,6^ who make up the largest proportion of users in some countries.^7,8^ The use of vapes is rapidly evolving in young people, including increases in use of disposable and nicotine-containing products,^9^ with access occurring through a range of social (e.g. peers) and commercial channels.^10,11^ In some countries, there is also evidence that young people are vaping more with products that have higher nicotine concentration.^12^

Globally, the increase in e-cigarette use among young people can be attributed to a range of interconnected social, environmental, commercial, and political determinants.^13^ For example, marketing and novel products have played a significant role in the normalisation of these products for young people, including positively shaping their attitudes towards initiation and social acceptance of e-cigarettes.^14–16^ This includes a range of flavours and novel product designs that continue to grow the tobacco industry’s customer base through attracting younger users who may never have engaged with tobacco cigarettes,^17,18^ and promoting a perception that these products are less harmful than cigarettes or could be considered healthy.^19,20^ Research also demonstrates the importance of social norms in the social acceptance and regular use of these products. Young people report that exposure to e-cigarettes in everyday settings creates an exaggerated perception that ‘everyone’ is using these products, and that they are an entrenched part of adolescent identity.^21^ Part of this normalisation process may also include a lack of understanding about the risks and harms associated with vaping, or that young people are aware of the harms but perceive they will be able to mitigate the harms from vapes, including through stopping vaping before these harms impact them personally.^21^ While young people are generally highly aware of the harms of cigarettes and hold negative views about the tobacco industry, they are less aware of the risks associated with the nicotine content of vapes,^22^ and the commercial tactics of the vaping industry (including that vaping companies are often owned in whole or part by Big Tobacco).^23,24^

Recognising these concerns, some governments have committed to increased restrictions on vaping to protect the health and wellbeing of children and young people. For example, in January 2024, the United Kingdom (UK) government announced policy measures to tackle youth vaping, including banning disposable vapes, restricting vape flavours and packaging, and powers to change how vapes are displayed in retail outlets.^25^ In Australia, the federal government has introduced legislation that seeks to implement some of the strongest regulatory restrictions on vapes in the world, including strict product standards; access to nicotine vaping products (with a concentration of 20 mg/ml) through participating pharmacies with access to under 18s only available with a prescription where state and territory laws allow;^26^ monitoring and preventing illegal importation and supply of non-prescription e-cigarettes; and raising public awareness about the health impacts of these products, alongside strong new measures to reduce smoking.^27,28^ Despite these important intentions to enact reforms in both Australia and the UK, the global risk to youth health from these products remains, with few other countries yet to take strong regulatory approaches to prevent their harms.^29^

Public support for preventive health policies has been identified as an important part of convincing governments about the need for such policies, and their subsequent implementation.^30^ Parents have been described as essential stakeholders in policy implementation relating to both tobacco and vaping – but are rarely surveyed about their attitudes towards regulatory responses to these products.^31^ Understanding parents’ views has been an important part of developing public health responses to harmful products, with parents demonstrating that they are important advocates in arguing for the increased regulation of these products to protect children.^32,33^ Engaging parents in discussions about the prevention of harms from vaping products is also important given that they may play a role in shaping young people’s vaping attitudes and behaviours,^34^ including as a risk factor for the uptake of vaping in children if a parent is an ‘ever-vaper’.^35^ While there is evidence in Australia and the United States that parents are supportive of a range of regulations aimed at controlling the marketing, public use, product formulation, and packaging of vapes,^33,36^ few qualitative studies have explored parents’ opinions, awareness, and observations of the range of factors that may contribute to the normalisation of vaping.^37^

The following study collected data in 2022 which was prior to significant changes in vaping regulatory reforms in Australia. At the time of data collection, Australia was facing challenges in enforcing its nicotine vape prescription-only access system, which aimed to restrict access to nicotine-containing vapes. Loopholes in this law allowed non-nicotine vapes to be marketed and sold widely, leading some retailers to sell nicotine-containing products under misleading claims of being nicotine-free. This regulatory environment, coupled with extensive advocacy and media attention, likely heightened parental awareness of vaping’s potential impact on children.

This study aimed to understand how parents conceptualised the factors that contributed to the normalisation of vaping for young people. Normalisation in the context of this study draws upon definitions from other areas of public health such as gambling,^38^ and is defined in this context as:

*The range of individual, socio-cultural, environmental, commercial, and political factors that may influence the initiation and regular use of vapes, and that shape the social acceptance of vaping (and vaping products) as an activity that is embedded and accommodated in everyday practices and routines.*

Three research questions guided the study:

*Research Question 1 (RQ1).* What are the range of factors that parents attribute as playing a role in shaping the normalisation of vaping for young people?
*Research Question 2 (RQ2).* How concerned are parents about the impact of vaping on the health and wellbeing of young people?
*Research Question 3 (RQ3).* Are parents aware of any efforts to raise awareness of the risks of vaping?

## Methods

### Approach

The data presented in this article were part of a larger study investigating Australian parents’ attitudes about the risks posed by addictive consumption industries (tobacco, alcohol, gambling, and vaping) to young people. The study utilised a qualitatively led online panel survey, which has been used in other studies to gain narrative insights into community attitudes about the practices of harmful industries.^39^ While these types of surveys elicit shorter textual responses than other more traditional methods of qualitative data collection, the information is still rich and is important in gaining insights from a broader and more diverse sample of the community.^40,41^ Researchers argue that there are a number of benefits associated with these types of qualitative studies, including geographical reach, anonymity, the ability to participate at a place and pace convenient to the participant, disrupting power relationships between researcher and participant, and engaging individuals in qualitative research that may not otherwise participate in face to face studies.^41^ Low-risk ethical approval was obtained from Deakin University (HEAG-H 158_2022).

### Sample and recruitment

Individuals were able to participate in the study if they identified as a parent of at least one child aged 11–17 years, living in Australia, and who could complete the survey in English. Soft quotas were used for gender and geographical locations across Australia. The proposed target sample size of around *n* = 500 participants was similar to other online qualitative studies about harmful industries,^39,42^ and aimed to collect enough information from a diverse group of participants to answer the study research questions.^43^ Recruitment of the sample was conducted through Qualtrics, who sent a link to potential participants who had signed up to a range of research panel companies. Online qualitative surveys are increasingly used by public health researchers to gather a broad range of insights into community attitudes and opinions about public health issues.^41^ When participants accessed the survey, they were provided with a description of the study and a downloadable Plain Language Statement. Participants consented to participation before starting the study and could withdraw from the study by closing their browser at any time. Participants received points for completing the survey, which could be redeemed for vouchers; this process was managed by the panel providers and not by the research team.

### Data collection

Data were collected over 4 weeks in December 2022, with the survey piloted with 30 parents to check for the quality of responses, interpretation of the questions, and any possible technical errors. Once the quotas had been achieved, Qualtrics performed an initial quality check of the data, and then the research team reviewed the data. The research team subsequently removed 47 participants due to unreliable data (such as nonsensical responses, responses inconsistent with the questions or entering random characters so that they could proceed to the next question), and new participants were recruited. We also screened all the responses from individuals under 35 and over 60 to ensure that the information that they gave about the age of their children was plausible. We subsequently removed 45 participants: this included, for example, an 18-year-old male who said that he had one male child aged 17 years.

Related to the data presented in this article, there were a range of discrete choice and open text questions. Discrete choice questions included age, gender, state of residence, level of education, and employment status; participants’ own vaping behaviour; whether they had engaged in vaping in front of their children; and whether they were aware if their child had used or tried to use vapes. They were also asked to rank how normalised vapes were for children (compared to tobacco, gambling, and alcohol). The *n* = 158 (34.7%) of parents who selected vapes as *the most* normalised were then asked to provide comment about why they selected this. Open text questions included the reasons young people might be interested in vaping, the main risks associated with vaping for young people, how children were being reached by marketing for vaping on social media platforms, and their attitudes about the use of flavours as a marketing strategy and how these might appeal to young people.

### Data analysis

Quantitative data were entered into Statistical Package for the Social Sciences (SPSS), and basic descriptive statistics were calculated relating to the socio-demographics. Qualitative data were analysed using six phases of reflexive thematic analysis which included familiarisation with the data through reading the text, challenging our assumptions about how we were interpreting the data, and considering different perspectives^44,45^. Preliminary codes and ideas were developed by ST, followed by team meetings where concepts were reflected upon and discussed. Coding was conducted across the data set in alignment with the research questions, with all authors engaged in reflective practices throughout this phase. The team collaboratively discussed the interpretation of the data and the construction of themes relating to overall patterns in the data, ensuring they were distinct from one another and accurately represented the data. Final themes were named and defined and reported using illustrative quotes.

## Results

### Socio-demographic and vaping characteristics

Parent socio-demographic and vaping characteristics are presented in Table 1. The sample was evenly distributed by gender, and most participants resided in the most populous Australian states of New South Wales (*n* = 125, 27.5%), Victoria (*n* = 108, 23.7%) or Queensland (*n* = 107, 23.5%). Over a third of participants were aged 35–44 years, and over a third were aged 45–54 years. The sample was highly educated, with 74.1% of the sample having at least some form of tertiary education (*n* = 337), and over half were employed full time (*n* = 259, 56.9%). Just under a fifth of participants reported using a vape in the last month (*n* = 70, 15.4%), and over half of all those who had vaped in the last month (*n* = 39, 55.7%) reported that their child or children had been present when they had used a vape. About 14% of the total sample reported that they were aware that their children had tried or currently used vapes (*n* = 64). This included the children of 21 participants who had previously used a vape themselves.

**Table 1:** Socio-demographic and vaping characteristics of parents (*n* = 455).

Gender	Frequency	Percentage
Male	234	51.4
Female	221	48.6
Age group	Frequency	Percentage
25–34	43	9.5
35–44	192	42.2
45–54	175	38.5
55–64	39	8.6
65+	6	1.3
State/territory	Frequency	Percentage
Northern Territory	4	0.9
ACT	6	1.3
Tasmania	14	3.1
South Australia	45	9.9
Western Australia	46	10.1
Queensland	107	23.5
Victoria	108	23.7
New South Wales	125	27.5
Education level	Frequency	Percentage
Below year 10	5	1.1
Year 10	36	7.9
Year 12	77	16.9
Certificate I, II, III, IV	87	19.1
Diploma/Advanced Diploma	62	13.6
Bachelor’s Degree	115	25.3
Graduate Diploma/ Graduate Certificate	15	3.3
Postgraduate Degree	58	12.7
Employment status	Frequency	Percentage
Working full-time	259	56.9
Work part-time or casually	98	21.5
Homemaker	58	12.7
Retired	13	2.9
Unemployed but looking for work	12	2.6
Full-time student	4	0.9
Other	11	2.4
Personally used in the last month	Frequency	Percentage
Yes	70	15.4
No	378	83.1
Prefer not to say	7	1.5
Has your child been present when you have used	Frequency (*n* = 70)	Percentage
Yes	39	55.7
No	31	44.3
Child’s use	Frequency	Percentage
Yes	64	14.1
No	388	85.3
Prefer not to say	3	0.7

Table 2 provides a summary of the key qualitative themes that were constructed from the data, with illustrative quotes from parents.

**Table 2:** Key themes about parents’ views about the normalisation of vaping for children and young people.

Theme	Subtheme	Illustrative quotes
Theme 1: Popularity and peer groups	Peer pressure	Peer pressure from friends who are doing it at school and outside of school. 52-year-old, Female, VIC Peer pressure from mates. 36-year-old, Female, NSW All their peers are trying and experimenting. 41-year-old, Male, QLD.
	Self-image and belonging	It’s become trendy to vape. 46-year-old, Female, NSW They are trying to fit in with the rest. 38 years old, Female, QLD Vaping is the current trend. It is seen as socially acceptable because everyone is trying it. (or it seems that everyone is). 40-year-old, Female, NSW
	A product associated with rebellion and peer acceptance.	Normal teenage rebellion and fitting in. 38-year-old, Female, WA The perception that it is cool and rebellious. 48-year-old, Female, QLD Rebellion – they know they are not allowed to do it. 41-year-old, Male QLD
Theme 2: The accessibility and availability of vaping products	Readily available	Vaping is convenient and easy to get. 31-year-old, Male, WA Vaping is much cheaper these days and you can buy them online. 48-year-old, Female, SA
	Cheap to buy and easy to hide	It’s cheaper and the teens can hide it. 47-year-old, Female, VIC They are cheap, small, easy to hide from parents or school and they don’t leave that residual smell like cigarettes do. 50-year-old, Male, NSW It’s becoming more socially acceptable, because it’s easy to access and its cheap. 52-year-old, Male, NSW
Theme 3: The influence of novel products and promotions	Flavours and the volume of ‘smoke’	Yes because of the fruity flavours. 30-year-old, Female, QLD. Vaping for young people - there is just a lot of different flavours and the amount of smoke you blow out I believe is very attractive for young people these days. 38-year-old, Male, VIC
	Promotions which specifically target young people	Vaping seems to be marketed towards kids. 34-year-old, Male, NSW Vape advertising targets young people. 43-year-old, Female, NSW It is advertised on social media. 37-year-old, Female, VIC
Theme 4: The perception that vapes were a healthier alternative to cigarettes or not harmful.	Perception vapes are a healthier alternative or less harmful than cigarettes	I am a little bit concerned of vaping, but not overly concerned because it is a better option than smoking cigarettes. 38-year-old, Male, VIC It’s a strong alternative to cigarettes. 39-year-old, Male, NSW Because it tastes good and is not seen as harmful. 50-year-old, Male, QLD They have the misconception that sucking in the vapour is no harm to them. 35-year-old, Female, QLD It’s the new cool thing to do and seen as healthier than smoking cigarettes. 41-year-old, Female, VIC Thinking as a healthy alternative to cigarettes. 46-year-old, Male, SA Under the assumption it’s a safer option than cigarettes. 48-year-old, Female, NSW Yes, vaping now is what smoking was 25 + years ago, and the dangers are vastly under publicised with it often being promoted as a healthy alternative and many people still believing it’s not bad for you. 38-year-old, Female, WA
	Young people lack understanding about the health risks of vapes	There is no concrete evidence of health risks. It comes in inviting flavours and looks cool. 41-year-old, Female, VIC The health risks that they don’t realise. 38-year-old, Female, SA So many people do it and there is no proven health risks yet. 41-year-old, Female, VIC It’s so new that there isn’t much studies done yet in relation to the health risks involved. 47-year-old, Female, VIC

### Theme 1: Popularity and peer groups

Many parents believed that peer pressure and influence was the main factor that prompted young people to try or use vaping. Parents wrote about the ‘*peer group pressure*’ that young people were experiencing to engage in vaping, describing that vaping was an activity that their children’s friends were regularly engaging in. Some parents believed that there was an expectation and pressure for children to engage in vaping with their friends to look *‘cool’* and *‘fit in with their peers’*. Because of this, parents perceived that this would spark young people’s curiosity *‘to see what it is like’*. Some parents specifically mentioned that engaging in vaping would contribute to young people being accepted by, and included in their peer groups:

*To be accepted by friends and look cool by following the trend. –* 39-year-old, Female, New South Wales.

Parents wrote about the importance of self-image and belonging to young people, and that engaging in vaping ensured that they fit in *‘with the ‘in’ crowd’* and were not left out of their social groups. There was broad concern about the extent to which vaping was becoming embedded within some social groups. Many parents discussed that vaping was a popular new trend that was cool, and easier to engage in (and hide) than smoking. They were concerned that peer pressure would lead their child to try vaping, and for it to become a regular activity in their friendship groups:

*Vapes are very dangerous and I’m concerned for my children when they are with their friends, i.e. peer pressure.* – 46-year-old, Female, Queensland.

Some parents commented that young people may also engage in vaping because it was a risky activity that could be seen as showing that they were rebellious or cool. Parents described that young people used vaping to *‘show off to their peers’, ‘impress their friends’*, and *‘be different e.g. rebel’*. Parents often linked school to rebellious behaviours, suggesting that young people were vaping at school, selling vapes at school, or vaping on the way to and from school:

*My daughter has told me that children the same age as her frequently vape on the school bus or at school, shopping centres. She talks about lots of kids she knows who use it. –* 32-year-old, Female, Victoria.

### Theme 2: The accessibility and availability of vaping products

Parents commented that vapes had become more socially acceptable and normalised for young people because they were highly accessible and available as compared to other products (such as cigarettes) – *‘they can’t get their hands on tobacco so they vape instead’*. Parents stated that vapes were readily available in local community settings, including being able to purchase them at the local corner shop, or that they could buy them online:

*Vaping is very popular among young people, it is advertised on social media and is perceived as being ‘cool’. The flavours of vaping fluid can be collected and shared and there are limited restrictions, which makes vaping very accessible.* – 37-year-old, Female, Victoria.

Parents perceived that this amplified the risks and acceptance of these products for young people, given that the *‘access is so easy’* and that they were *‘something kids can get their hands on’*. They commented that the accessibility of vapes also created an exaggerated perception for young people that most other young people were vaping, and that *‘everyone is doing it’*. Parents commented that vapes were also much cheaper to buy than cigarettes. The inexpensive nature of these products, and the lack of smell meant that the consumption of these products was easier to conceal from their parents:

*Cheaper and no lingering smell makes it easier to get and easier to hide. –* 41-year-old, Female, Victoria.

### Theme 3: The influence of novel products and promotions

Many parents commented that vaping had become more normalised for young people because the product had distinct sweet flavours and branding that was highly appealing to young people. This included that vapes *‘taste like lollies’*, and had *‘appealing flavours (e.g. fruit, candy, dessert)’*. They stated that the flavours of vapes made the product seem more ‘*fun*’ and increased young people’s curiosity to try these products. Many parents suggested that there was something particularly appealing about the way vapes were marketed to young people, with a few describing vaping as fashionable:

*Using appealing flavours and colours normalise this for kids they see it in a positive light and it appeals to them like lollies.* – 41-year-old, Male, Victoria.

Parents also suggested that young people would be interested to try something new and *‘see what will happen to them when they intake it*’. These product features added to young people’s curiosity to try the range of flavours that were available, with the marketing of flavours central to the promotion of vapes:

*More socially acceptable, tastes better than cigarettes, huge range of desirable flavours, cheaper than cigarettes.* – 45-year-old, Female, Tasmania.

Parents also wrote about the novel range of promotions that were used to sell vapes, particularly in relation to marketing on social media platforms. Parents stated that vapes were deliberately marketed towards young people – ‘*Vape advertising targets young people’*. Products were promoted on sites such as TikTok, and parents were concerned that young people would be particularly influenced by this type of marketing, which glamourised vaping while also promoting that they were less harmful than cigarettes:

*Advertised as a cool thing and healthier than ciggies. –* 52-year-old, Female, Queensland.

### Theme 4: A perception that vapes were a healthier alternative to cigarettes or not harmful

Finally, parents perceived that many young people believed that vapes were either harmless, or less harmful than cigarettes, and were *‘. . . targeted as fun and better for you than smoking’* and were largely unaware of the risks associated with these products. Some attributed this to the marketing of vapes which were advertised as a way to quit smoking or a healthier alternative to cigarettes. While some parents believed that these products were less harmful than cigarettes, other parents were very critical of messages that vapes were less harmful than cigarettes stating that there was a perception that vaping is *better than smoking, and it’s not’*. Parents stated that these products were dangerous, containing harmful chemicals, and referred to vapes as *‘poison’*:

*I think it’s becoming more popular as it is a supposed healthier replacement for cigarettes which i think is the biggest lie ever told. I reckon it’s the most dangerous product out there for your health.* – 50-year-old, Male, Queensland.

Parents were clearly concerned about a variety of health harms associated with vapes. These included concerns about addiction, the impact on young people’s lung health, and cancer. A few parents stated that despite vapes being widely available, there was limited evidence about the risks (and safety) of these products. When discussing the risks associated with vapes, a few parents implied that the social acceptability and perception that these products were ‘cool’ outweighed young people’s understanding of the risks associated with these products:

*Frightened and worried about them getting into a bad habit that they will get addicted (to).* – 52-year-old, Male, South Australia.

*Can lead to addiction, more accepted in social circles as it looks cool & promoted to be a safer option which it is not, not good for lungs & can cause cancer.* – 48-year-old, Female, New South Wales.

Even when there was widespread attention in the media about the potential risks associate with vaping, young people still wanted to try these products:

*Vaping is seen a lot in the media, via newspapers, social media, news reports etc usually associated with a negative aspect however, teens are still seeing it and wanting to try despite the negatives.* – 40-year-old, Female, New South Wales.

One parent stated that the misperceptions around vaping, and the use of these products, had led their child’s school to send home a newsletter warning parents about vaping risks for children’s health:

*Because this is very popular amongst the children at my child’s school and there has been school newsletters explaining why vaping is not good for their health. –* 52-year-old, Female, Victoria.

Others noted that the initial promotions of vapes as a cheaper, healthier alternative to cigarettes, coupled with the lack of evidence about harms, and the more pleasant taste and smell of these products had created a health halo whereby young people thought they were less risky and harmful than traditional cigarettes:

*It smells better than smoking and the dangers are less visible.* – 34-year-old, Female, New South Wales.

## Discussion

**Figure 1:**
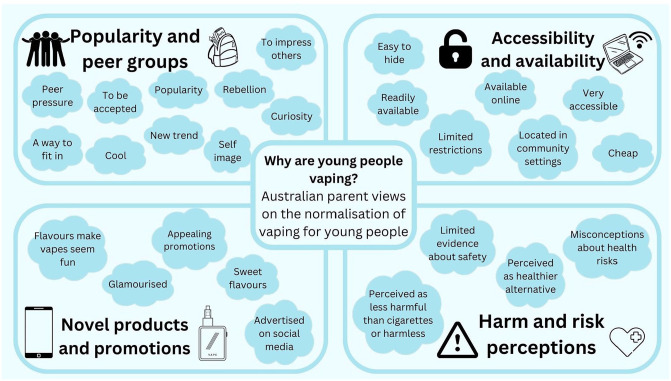
Parent’s perceptions of the factors contributing to the normalisation of vaping

This study aimed to investigate Australian parents’ views about the factors contributing to the normalisation of vaping for young people. The study was conducted in the lead-up to Australia’s strong regulatory approach to vaping which seeks in part to prevent children from widespread exposure to vaping products. This study provides important information for public health organisations about parental awareness of the normalisation of vaping and how these views could be used to both increase understanding about the normalisation of vaping for children, and to advocate for policy reforms. Documenting parents’ concerns about the normalisation of vaping provides critical evidence that can be used to inform policymakers about the demand for action to protect children from potential harm. Figure 1 provides an overview of the range of factors that parents in this study thought were contributing to the normalisation of vaping for young people.

The results from this study demonstrated that parents attribute the normalisation of vaping to a combination of social meanings and contexts; the availability and accessibility of vapes – including the price of these products and the ability of children to hide them from parents; the marketing tactics of the vaping industry; and the subsequent perceptions that vapes are a less harmful product than cigarettes or harmless. As has been observed in other studies^20,33–35^ parents were aware that vaping is becoming an increasing phenomenon in young people’s lives, they have strong recognition of the various features that make vaping appealing for young people (including flavours and novel product design), and are concerned about the potential risks associated with these products for young people.

However, despite acknowledging the role of novel products and marketing in the normalisation of vaping, only a few parents directly attributed this to the tactics of the vaping industry, or mentioned that vapes were illegal for young people to purchase and consume. Rather, the default perspective for some parents was that peer pressure (including from a few rebellious children leading others down the wrong path), social group identity and belonging, and curiosity were the main reasons why young people vaped. Parents were less aware that vaping industry marketing often frames vaping as central to youth identity and belonging, a strategy that research suggests contributes to youth curiosity to try vaping through exposure to advertising.^1^ Similarly, parents perceived that vaping is considered a less harmful alternative to smoking, or that there is a lack of evidence about harms, but very few parents directly mentioned how the vaping and tobacco industries exploit this framing in their advertising and promotion to reassure users that vaping products are safe.^46,47,47^ These findings suggest that rather than focusing on young people’s behaviour, public health organisations could focus more explicitly on the role of the vaping industry in positioning and promoting products in a way that appeals to children and young people. One strategy may be to highlight the links between vaping and the tobacco industry – drawing parallels between these tactics, including the strategies used to appeal to children, and how these products are being used by the tobacco industry to *‘reinvent itself by introducing a portfolio of new products’*^48^ (p. 334)

Parents often mentioned the role that the school environment plays both as a space in which vaping may occur, but also as a key partner in educating children about the harms associated with vaping. Some acknowledge that schools had already communicated the risks associated with these products to parents. Ensuring schools are not left to ‘solve’ youth vaping through education and behavioural interventions alone requires strong policy responses that address upstream measures such as vape access and supply. School-based vaping interventions can then amplify and support these broader policy measures.^49^ Leveraging strong parental and school concern is also an important public health advocacy tool and prioritising these voices in communications with elected official and through the news media is crucial.^50^

Parents acknowledged that vapes were relatively easy to access and easy to hide from parents and those in positions of authority (such as teachers). This meant that vapes were easier to access and use than other harmful products such as alcohol or cigarettes. These views are reflective of the poor implementation and subsequent enforcement of the nicotine vape prescription-only access system in Australia at the time of data collection. Loopholes in this law permitted non-nicotine vapes to be sold as commercial goods at retailers across Australia. However, retailers were openly selling nicotine-containing vapes under the guise that they did not contain nicotine. The reforms introduced in early 2024, and those scheduled for implementation in 2025, address this significant loophole that has severely hampered the effective implementation and enforcement of the prescription-only access framework.^51^

## Limitations

The study was conducted in the lead-up to Australia’s strong regulatory approach to vaping which aims in part to prevent children from widespread exposure to these products. At the time that this study was conducted there was extensive advocacy and media attention about vaping, which may have meant that parents in this study were more aware of vaping and any potential impact on children. While a range of checks on the data were performed to determine the individuals who participated in the study were providing reliable information about being parents of children, the anonymous nature of these types of surveys means there is no way of being completely sure that all of the individuals who participated in the study had children in the age range stipulated in the study inclusion criteria. This study only recruited parents, and did not seek the views of a range of caregivers who may have primary responsibility for children.

## Conclusion

Vapes have developed speedily from a novel and little-known invention to products and behaviours that have been widely adopted in many countries far beyond the initial purpose of providing a possible cessation mode for adult smokers. Parents are understandably concerned about the normalisation of vaping among young people, which is occurring at a time when health authorities should be building on the decline in smoking in both adults and children. Harnessing parental concern and increasing their understanding of the key role the vaping and tobacco industries play in driving the alarming increase in youth vaping are useful strategies to securing strong regulation of vaping products. Firm action is needed to shift industry-friendly communications and narratives away from peer-pressure and teen rebellion to focus on the role of vape product design and promotion and ease of vape access. This should include comprehensive and well enforced legislation on marketing of vapes and ensuring that information about health and other aspects of vaping comes from health authorities, not commercial interests or those they fund.
